# Contrast-enhanced Perfusion Measurements in Patients with Active Crohn’s Disease Using Sonazoid

**DOI:** 10.1055/a-2830-9879

**Published:** 2026-04-08

**Authors:** Kim Nylund, Fredrik Sævik, Odd Helge Gilja

**Affiliations:** 1 60498 National Center of Ultrasound in Gastroenterology, Haukeland University Hospital, Bergen, Norway; 2 1658 Department of Clinical Medicine, University of Bergen, Bergen, Norway; 3 114318 Department of Medicine, Sørlandet sykehus HF Arendal, Arendal, Norway

**Keywords:** Crohn’s disease, hemodynamics/flow dynamics, disease activity, contrast agents

## Abstract

**Purpose:**

This study aimed to assess the feasibility of dynamic contrast-enhanced ultrasound with Sonazoid, its correlation with disease activity, its ability to differentiate between remission/mild activity and moderate/severe activity, and if a region of interest including the mucosa and submucosa was different compared to one encompassing the entire wall.

**Materials and methods:**

We prospectively studied 48 patients with Crohn’s disease who underwent colonoscopy and dynamic contrast-enhanced ultrasound with Sonazoid from 2015 to 2019. We compared the local simple endoscopic score for Crohn’s disease in the most affected area with parameters from Vuebox, including peak enhancement, wash-in and wash-out area under the curves, wash-in and wash-out rates, and wash-in perfusion index. Region of interest-1 (ROI-1) included all wall layers, and region of interest-2 (ROI-2) included the mucosa and submucosa. Linear data and normalised data were used. Technical failure or a quality of fit value of<80% was considered a failed examination.

**Results:**

The feasibility of the dynamic contrast-enhanced ultrasound examination was 73%. No significant findings were noted in the linear data ( *p* >0.05). In the normalised data in both ROI-1 and ROI-2, the local simple endoscopic score for Crohn’s disease correlated significantly with peak enhancement ( *r* =0.38 and *r* =0.35), wash-in rate ( *r* =0.34 and *r* =0.34), and wash-in perfusion index ( *r* =0.40 and *r* =0.37) and with wash-in area under the curve ( *r* =0.36) in ROI-2. In patients with moderate/severe disease peak enhancement, wash-in area under the curve, wash-out area under the curve, wash-in rate, and wash-in perfusion index were significantly higher in both region of interests ( *p* <0.05). All parameters were significantly different in ROI-1 and ROI-2 ( *p* <0.05).

**Conclusions:**

Dynamic contrast-enhanced ultrasound parameters can differentiate between remission/mild and moderate/severe activity in Crohn’s disease, but the method has relatively low feasibility.

## Introduction

Crohn’s disease (CD) is a chronic inflammatory disease of the bowel. To avoid long-term complications, active inflammation should be treated despite symptomatic control. 1​ Gastrointestinal ultrasound (GIUS) is an attractive modality for monitoring disease activity, as it is well tolerated by patients, has no ionising radiation, is inexpensive, and can therefore be repeated as frequently as needed to assess the effects of medical therapy. 2​

The main parameter used to assess disease activity with GIUS is the bowel wall thickness (BWT), 2​ but a thickened bowel wall is also associated with fibrosis. 3​ ​4​ Colour Doppler aids in detecting inflammation and is included in all ultrasound activity scores. 5​ 6​ 7​ 8​ ​9​ However, the sensitivity of the colour Doppler is limited by depth. 10​ While a mid-range linear probe can produce images down to 8–10 cm, the colour Doppler is typically limited to 4–5 cm. 10​ Colour Doppler is useful because the inflammatory activity of CD causes increased microvessel density. 5​ Contrast-enhanced ultrasound (CEUS) using SonoVue has been shown to correlate with microvessel density 6​ and endoscopic inflammatory activity in CD. 7​ 8​ 9​ 10​ ​11​ Results are conflicting regarding blood flow, but most CEUS studies show increased bowel wall contrast enhancement in active CD. This was recently confirmed in a meta-analysis, 12​ but there is a considerable variation in results, questioning the applicability of CEUS in CD.

Recent studies have indicated that CEUS is not necessary in patients with mild disease but could be useful for distinguishing moderate and severe diseases from mild disease. 7​ ​10​ Selecting a region of interest (ROI) for analysis in a thin bowel wall is technically demanding. During dynamic contrast-enhanced ultrasound (DCE-US), the region becomes even harder to track over time because it is more susceptible to noise from gas in the bowel lumen. Nylund et al. found that healthy volunteers had the greatest variability in all perfusion parameters. 3​ Furthermore, in the GI tract, the mucosa and submucosa are significantly better perfused than the proper muscle. 13​ It would therefore be of interest to investigate whether focusing the ROI on the mucosa and submucosa could provide better results.

SonoVue was used in all the studies previously cited. Sonazoid is another ultrasound contrast agent that has been clinically available since 2006, but only in some countries. 14​ Its main use is for differentiating liver lesions due to its long-lasting stability in the liver and its tendency to be taken up by Kupffer cells of the liver sinusoids. 15​ The microbubbles of Sonazoid have a much more uniform and narrow distribution range of the volume median diameter (SonoVue 8.0±0.9 µm vs Sonazoid 2.6±0.1 µm). 14​ This could make quantification easier since the transducer could detect all microbubbles, and you can avoid attenuation from microbubbles outside the frequency range.

There are, however, very few studies in which Sonazoid has been used for DCE-US and no human studies have examined patients with inflammatory bowel disease.

This study aimed to examine the feasibility of DCE-US with Sonazoid in patients with active CD, assess its correlation with endoscopic activity, determine its ability to differentiate remission/mild disease activity from moderate to severe disease activity, and examine if the parameters from the entire GI wall differ from those of the mucosa and submucosa.

## Materials and methods

### Study design and patients

This was a single-centre, prospective, cross-sectional observational study from 2015 to 2019 at our hospital with consecutive recruitment. Inclusion criteria were ileocolonoscopy performed in patients with CD. Exclusion criteria were no activity on ultrasound (BWT<3 mm), age<18 years, ongoing gastroenteritis, isolated disease in the upper GI tract, and balloon dilatation during ileocolonoscopy or previous colectomy. Patients with partial colectomy or ileocecal resection were included. The patients were a part of a larger cohort that also included patients in remission, but those patients were not part of this study. The indication for endoscopy varied between primary diagnosis, exacerbation, treatment evaluation and surveillance. All patients with known or suspected CD were considered eligible, except those referred specifically for balloon dilatation of a stenosis.

### Clinical and biochemical measurements

Clinical data were collected on the day of the GIUS, including the Harvey Bradshaw index. 19​ The examiner recorded patients’ gender, age, weight, and height, as well as the known extent of CD, ongoing drug treatment or prior surgery. The patient was categorised according to the Montreal classification. 20​

Depending on when the patient was recruited relative to the endoscopy, blood and stool samples were collected within±1 week of the GIUS examination. Blood samples were analysed for serum haemoglobin (g/dL), leucocyte count (10 ^9^ /L), platelet count (10 ^9^ /L), C-reactive protein (mg/L) and serum albumin (g/L). Stool samples were also collected, and faecal calprotectin was measured (µg/g). The information was collected from the patient or the patient’s journal.

### Gastrointestinal ultrasound

The ultrasound examination was performed as close to the endoscopy as possible (±14 days), without being on the same day or during bowel preparation, as bowel preparation causes the bowel lumen to collapse, which could make standardisation more difficult. The examination was conducted by the main investigator, who had 6 years of experience with GIUS at the study’s outset. There was no change in medical treatment between the ultrasound and endoscopic examination. The examiner was blinded to the endoscopy results, but not to the patients’ Montreal classification or symptoms.

A Logiq E9 ultrasound scanner (GE, Milwaukee, USA) was used in the study, along with a curvilinear probe (C1-6, 1–6 MHz) and a linear probe (9L), 5.5–9 MHz. We used an optimised preset for bowel on the 9L probe. The examination was performed after an overnight fasting. The curvilinear probe was used for an overview and to examine the rectum, while the 9L probe was used for a systematic examination of the bowel. First, the terminal ileum was scanned, and then the colon from the ascending colon to the sigmoid colon. The proximal small bowel was not examined because it is beyond the reach of ileocolonoscopy. The data from the most affected segment, defined as the thickest bowel wall segment on GIUS, is used in this study.

The terminal ileum and all the segments of the large bowel were scanned in their full length to avoid missing skip lesions with the C1-6 probe for the rectum and the 9L probe for the rest of the bowel. The BWT was measured in the anterior bowel wall from the interface between the serosa and the proper muscle to the interface between the mucosa and the lumen.

Colour Doppler measurements were performed with the linear 9L probe with a velocity scale of 5 cm/s to detect the vessels in the GI wall. The examination was performed during breath-hold. The gain was increased until flash artefacts appeared and then reduced to the level at which the artefacts disappeared. BWT, colour Doppler, stratification, fatty wrapping, folds in the proper muscle, thickening of the muscular mucosa, compressibility, and the presence of complications such as stenosis and fistulas were recorded for each segment. Further details and definitions of different parameters can be found in the Appendix section.

### Dynamic contrast-enhanced ultrasound: Examination and analysis

DCE-US was performed on the most affected bowel segment. A venous catheter (20 Gauge) without a filter was inserted into a vein in the cubital fossa of the left arm and connected to a three-way interconnector. After mixing Sonazoid (GE Healthcare, Norway) according to the drug description, 1 mL of Sonazoid was injected as an intravenous bolus, followed by a flush of 10 mL of saline.

**Fig. 1:**
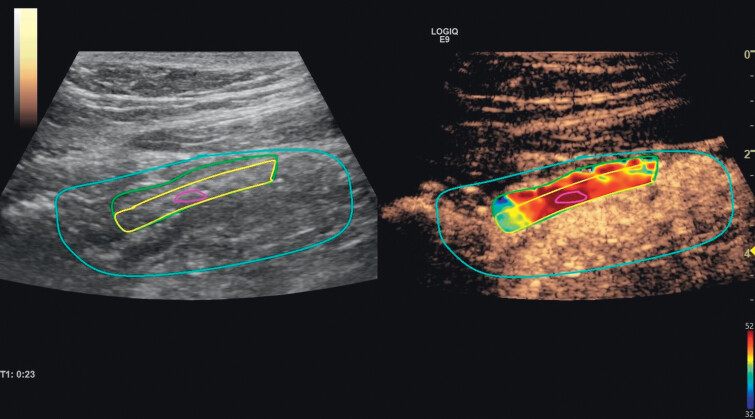
In the left image, a B-mode image of an inflamed terminal ileum is shown with several different regions drawn in various colours. The blue region represents the “area of interest” and is the image region that will be stabilized during the motion correction. All the other regions need to be drawn inside this area of interest, and it is drawn around the bowel segments as this will move during respiration, even if the investigator keeps the transducer fixed. The green region is drawn in the anterior wall of the bowel and includes the whole bowel wall (ROI-1). The yellow region is drawn within ROI-1 but only includes the mucosal and submucosal layer. The right image frame includes the contrast information, and here a “heatmap” of the contrast enhancement is shown where blue means low contrast-enhancement and red means high contrast enhancement. The final purple region is drawn within such a “hot spot” in the heat map. This represents an area of high contrast-enhancement (most likely a vessel) and is used for normalisation of the intensity data. To avoid attenuation effects, this hotspot was chosen at the same depth as and within the bowel wall. (ROI, region of interest.)

**Fig. 2:**
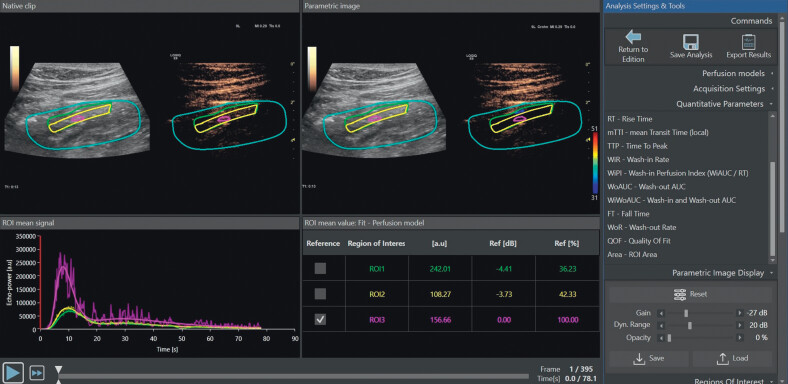
Screenshot of the user interface in Vuebox after analysis has been performed. The upper left image is the native clip while the upper middle image is the parametric image corresponding to Fig. 1 . In the lower left image, three curves are shown corresponding to the different regions of interest (ROIs) drawn within the blue area of interest. The purple curve represents the hotspot (ROI-3). The green curve represents ROI-1 and the yellow curve represents ROI-2. As expected, the amplitude of the contrast-enhancement within the hotspot is much higher than within the other two regions. In the middle lower image, the data for the PE is shown for all ROIs including the scaled values (column called Ref [%]). In the lower part of the image, a slide ruler can be moved around to play through the whole DCE-US video. The video in this image is only 78.1 seconds since the first part before the time of contrast arrival is cut from the video as a part of the analysis. Finally, in the right part of the image, action buttons for saving and exporting data are shown together with a list of all the DCE-US parameters. Not every parameter can be seen on the list of this screenshot as there is not enough room on the screen. (DCE-US, dynamic contrast-enhanced ultrasound; PE, peak enhancement.)

For the analysis of the 90-second contrast cine loop, Vuebox (Version 7.2, Bracco, Geneva, Switzerland) was used. Digital imaging and communications in medicine (DICOM) video files were exported from the Logiq E9 scanner and analysed on a separate computer after being uploaded. The Vuebox calibration for a Logiq E9, C1-6 ultrasound probe was used. Image data are log-compressed. Vuebox can, through approximation, relinearise the data from exported DICOM files. Two ROIs were used in the GI wall. ROI-1 included the entire wall, while ROI-2 included the mucosal and submucosal layers ( Figs. 1 and 2) . The parameters are shown in Table 1 .

**Table:** **Table 1** Perfusion parameters derived from the time–intensity curve (TIC) in a region of interest (ROI) in Vuebox

Parameters	Abbreviations	Definition
Time of arrival ^a^	ToA	Time when contrast enters the tissue
Peak enhancement ^b^	PE	The maximum intensity of the TIC
Rise time ^a^	RT	Time from ToA to PE
Fall time ^a^	FT	Time from PE to a point at the *x* -axis where the minimum slope tangent crosses
Mean transit time ^a^	MTT	Average time for the blood to pass through the ROC
Wash-in area under the curve ^b^	WiAUC	Area under the TIC from the time of arrival to the PE
Wash-out area under the curve ^b^	WoAUC	Area under the TIC from PE to the end of the curve
Wash-in/wash-out area under the curve ^b^	WiWoAUC	The total area under the TIC
Wash-in rate ^c^	WiR	The maximum slope of the TIC represented as a tangent at the ascending part of the curve
Wash-out rate ^c^	WoR	The minimum slope of the curve represented as a tangent at the descending part of the curve
Wash-in perfusion index	WiPI	WiAUC/RT
Quality of fit	QoF	The percentage of fit between the measured data and the model

^a^ Seconds (s).

^b^ Arbitrary units (a.u.).

^c^ a.u./s.

Further details on the examination and analysis are shown in the Appendix section.

### Ileocolonoscopy

Ileocolonoscopy was routinely performed at the endoscopy unit in our hospital. The examiners were blinded to the GIUS results, but not to the patients’ Montreal classification or symptoms. All examiners are taught and practice the use of the Simple Endoscopic Score of Crohn’s disease (SES-CD) at the hospital. 21​ SES-CD was recorded for all bowel segments, including the terminal/neo-terminal ileum, ascending colon, transverse colon, left colon, and rectum when reached by the endoscope. For comparison with DCE-US, the local SES-CD of the most affected segment, as defined on GIUS, was used. Overall, a SES-CD of≤2 was considered as remission, 3–6 as mild activity, 7–15 as moderate activity, and>15 as severe activity. Failure to examine proximal to a stenosis was not considered an exclusion criterion.

### Statistics

The data were analysed using descriptive statistics, presented as mean and standard deviation if normally distributed, and median and interquartile range if not. Kappa statistics were used to investigate the agreement between ultrasound and endoscopy for disease activity. Pearson’s or Spearman’s Rank correlation coefficient was used to assess dependence between variables, depending on whether they were normally distributed. Student’s *t* -test was used to assess independent, continuous variables with a normal distribution, and the Mann–Whitney *U* test was used when the data were not normally distributed. The paired sample *t* -test and the Wilcoxon signed-rank test were used to compare dependent variables. To examine diagnostic efficacy, a receiver operating characteristic (ROC) analysis was performed, and the area under the ROC curve (AUROC) was calculated. Youden’s index was used to find the optimal cut-off. The significance level was set at *p* <0.05. IBM SPSS software version 29 was used for the calculations.

## Results

Altogether 48 patients had disease activity in one or more bowel segments defined as a BWT of>3 mm in the inclusion period. Characteristics of patients with no activity or mild disease and patients with moderate to severe disease on endoscopy are shown in Table 2 .

**Table:** **Table 2** Patient characteristics in a study on bowel perfusion with Sonazoid in CD

Variables	Local SES-CD<7 ( *n* =36)	Local SES-CD≥7 ( *n* =12)
Gender (male/female)	21/15	3/9
Age, median (range)	40 (19–74)	41 (19–78
BMI, mean (SD; kg/m ^2^ )	25.4 (3.9)	25.3 (5.1)
Endoscopic remission, ^a^ yes/no	9/36	0/12
Age of onset diagnosis		
<16 y	5	2
16–40 y	24	7
>40 y	7	3
Disease location		
Ileum	13	4
Colon	6	2
Ileocolic	13	5
Upper GI	4	1
Disease type		
Non-stenosing, non-fistulating	7	1
Stenosing	15	8
Fistulating disease	9	1
Perianal disease	5	2
Previous surgery	18/36	5/12
Simple index (median/range)	3 (0–12)	5 (1–20)
Main drug at baseline		
None	6	0
Budesonid	3	1
Azathioprine	3	1
Purinethol	0	1
Infliximab	14	3
Adalimumab	5	4
Vedolizumab	3	2
Certolizumab	2	0

Abbreviations: BMI, body mass index; CD, Crohn’s disease; GI, gastrointestinal; SD, standard deviation; SES-CD, simple endoscopic score for Crohn’s disease.

^a^ A SES-CD of≤2.

### Endoscopy

Nine of the patients were in endoscopic remission, 27 had mild, 9 moderate and 3 severe endoscopic activity according to the local SES-CD. Overall, the SES-CD was 6.4±4.9 while the local SES-CD in the worst affected segment on ultrasound was 4.5±3.1. In total, there were 12/48 patients with a SES-CD of≥7 in the worst affected segment.

### Clinical and biochemical tests

Except for platelets ( *p* =0.043), there were no significant differences for any of the parameters between patients without endoscopic activity or with mild disease compared to patients with moderate to severe disease. The results are summarized in Table 3 .

**Table:** **Table 3** Clinical and biochemical tests in a study on bowel perfusion with Sonazoid in Crohn’s disease

Test	No or mild activity. Local SES-CD<7 ( *n* =36)	Moderate or severe activity. Local SES-CD≥7 ( *n* =12)	*p* -Value
Harvey bradshaw index ^a^	3 (6)	5 (5)	*p* =0.063
Haemoglobin (g/dL) ^b^	14.0±1.6	13.3±1.2	*p* =0.215
Leucocyte count (×10 ^9^ /L) ^a^	5.2±2.3	5.3±1.9	*p* =0.871
Platelet count (×10 ^9^ /L) ^a^	265±52	309±86	***p*** **=0.043**
Albumin (g/dL) ^a^	44.1±3.9	42.0±3.9	*p* =0.107
CRP (mg/L) ^b^	4 (11)	6 (9)	*p* =0.859
Faecal calprotectin (mcg/g) ^b^	118 (326), *n* =25	343 (797), *n* =10	*p* =0.529

Abbreviations: CRP, C-reactive protein; SES-CD, simple endoscopic score for Crohn’s disease.

^a^ Median and interquartile range. The Mann Whitney- *U* test.

^b^ Mean and standard deviation. Student’s *T* -test.

### Conventional ultrasound parameters

The bowel wall was significantly thicker (5.7 [±1.5] vs 6.8 [±1.8] mm, *p* =0.042), less compressible ( *p* =0.043) and had a higher stenosis score ( *p* =0.044) in segments with moderate to severe endoscopic disease activity in the same bowel segment compared to patients without or with mild activity. None of the other ultrasound parameters were significantly different as shown in Table 4 .

**Table:** **Table 4** Conventional ultrasound parameters in CD patients without and with mild endoscopic activity compared to patients with moderate to severe endoscopic activity

Parameters	Remission or mild disease (SES-CD<7), *n* =36	Moderate or severe disease (SES-CD≥7), ( *n* =12)	*p* -Value
BWT (mm) ^a^	5.7±1.5	6.8±1.8	**0.042**
Mucosa (mm) ^a^	1.6±0.7, *n* =31	1.9±0.4, *n* =12	0.227
Submucosa (mm) ^a^	2.4±1.0, *n* =31	3.1±1.6, *n* =12	0.133
Proper muscle (mm) ^a^	1.5±0.6, *n* =31	1.7±0.6, *n* =12	0.291
Length of affection (cm) ^a^	7.1±5.3	6.6±2.9	0.754
Colour Doppler score ^b^	13/8/13	2/5/5	0.309
Colour Doppler (no/yes) ^c^	13/21	2/10	0.156
Stratification score ^b^	21/9/4	8/3/1	1.000
Stratification loss (no/yes) ^c^	23/13	8/4	0.576
Fatty wrapping (no/yes) ^c^	26/8	6/6	0.143
Folded proper muscle (no/yes) ^c^	21/14	5/7	0.326
Thickened MM (no/yes) ^c^	24/10	10/2	0.472
Compressible (yes/no) ^c^	18/16	2/10	**0.043**
Any complications (no/yes) ^c^	18/16	3/9	0.176
Fistula (no/yes) ^c^	29/5	11/1	0.577
Stenosis score ^b^	21/12/1	4/5/3	**0.044**
Stenosis (no/yes) ^c^	21/13	4/8	0.107

Abbreviations: BWT, bowel wall thickness; CD, Crohn’s disease; SES-CD, simple endoscopic score for Crohn’s disease.

^a^ Student’s *t* -test.

^b^ Fischer–Freeman Halton's exact test.

^c^ Fischer’s exact test.

### Dynamic contrast-enhanced ultrasound

Of the 48 patients with disease activity on GIUS, three patients refused a contrast examination, two examinations failed due to technical reasons and in 10 patients the analysis was not acceptable due to bowel motion or poor image quality with a quality of fit value of<80%. Feasibility was 33/45 (73%).

**Fig. 3:**
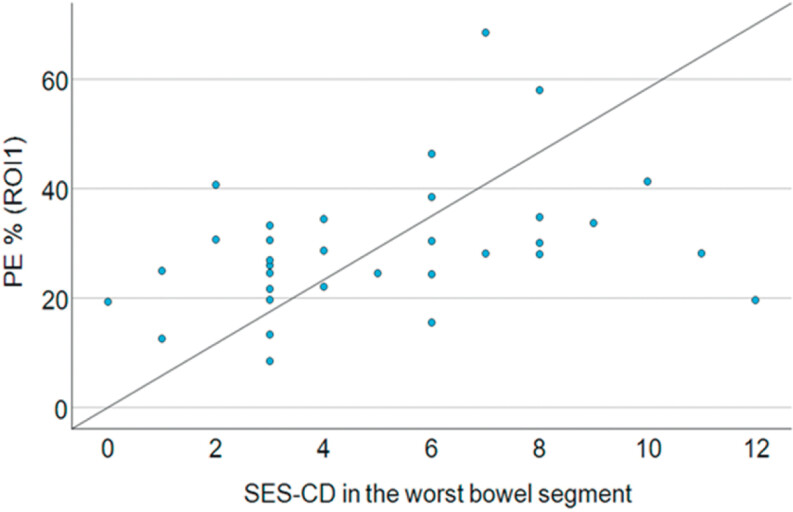
In the scatterplot, the normalised peak enhancement (PE) is compared to simple endoscopic score of Crohn’s disease (SES-CD) in the same bowel segment.

In the remaining 33 patients, there was no correlation between the segmental SES-CD and the linear DCE-US parameters, neither in ROI-1 nor in ROI-2. For the normalised parameters, there was a correlation between local SES-CD in ROI-1 for PE ( *r* =0.38, *p* =0.028), Fig. 3 , wash-in rate (WiR; *r* =0.34, *p* =0.050) and WiPI ( *r* =0.40, *p* =0.021). For ROI-2, PE (0.35, *p* =0.047), wash-in area under the curve (WiAUC; 0.36, *p* =0.041), WiR ( *r* =0.34, *p* =0.050) and WiPI ( *r* =0.37, *p* =0.032) correlated.

There were 10/33 patients with moderate or severe activity, and there was no significant difference between the patient groups for any of the linear data for ROI-1 or ROI-2 (See the Table in Appendix). For the normalised data, there was a relatively higher PE, WiAUC, wash-out area under the curve (WoAUC), WiWoAC, WiR and WiPI for both ROI’s (See Table 5 ).

**Table:** **Table 5** Scaled DCE-US parameters in a study on bowel perfusion with Sonazoid in CD

Parameters	ROI-1 ^a^			ROI-2 ^a^		
	Remission or mild disease (SES-CD<7) ( *n* =23)	Moderate or severe disease (SES-CD≥7) ( *n* =10)	*p*	Remission or mild disease (SES-CD<7; *n* =23)	Moderate or severe disease (SES-CD≥7) ( *n* =10)	*p*
PE (%) ^b^	25.0 (11.1)	31.9 (17.4)	**0.034**	32.0 (15.4)	41.7 (24.5)	**0.016**
WiAUC (%) ^b^	29.0 (20.6)	41.8 (35.3)	**0.038**	38.4 (20.9)	54.9 (39.6)	**0.042**
WoAUC (%) ^c^	36.1 (16.3)	52.0 (24.2)	**0.034**	44.7 (20.4)	64.4 (30.0)	**0.035**
WiWoAUC (%) ^c^	34.5 (14.2)	49.2 (21.0)	**0.025**	42.9 (18.4)	61.1 (25.5)	**0.027**
WiR (%) ^c^	22.6 (15.8)	33.1 (15.8)	**0.024**	29.7 (15.0)	43.1 (18.4)	**0.035**
WoR (%) ^c^	22.5 (13.3)	30.7 (18.0)	0.152	29.5 (19.5)	40.6 (22.6)	0.161
WiPI (%) ^b^	25.4 (10.4)	31.7 (17.0)	**0.025**	32.5 (15.4)	41.8 (24.5)	**0.014**

Abbreviations: CD, Crohn’s disease; DCE-US, dynamic contrast-enhanced ultrasound; PE, peak enhancement; ROI, region of interest; WiAUC, wash-in area under the curve; WiPI, wash-in perfusion index; WiR, wash-in rate; WoAUC, wash-out area under the curve; WoR, wash-out rate.

^a^ ROI-1 includes ultrasound layers corresponding to the mucosa, submucosa and the proper muscle. ROI-2 includes the ultrasound layers corresponding to the mucosa and submucosa.

^b^ Median (Interquartile range), Mann–Whitney *U* -test.

^c^ Mean (standard deviation), Students *t* -test.

**Fig. 4:**
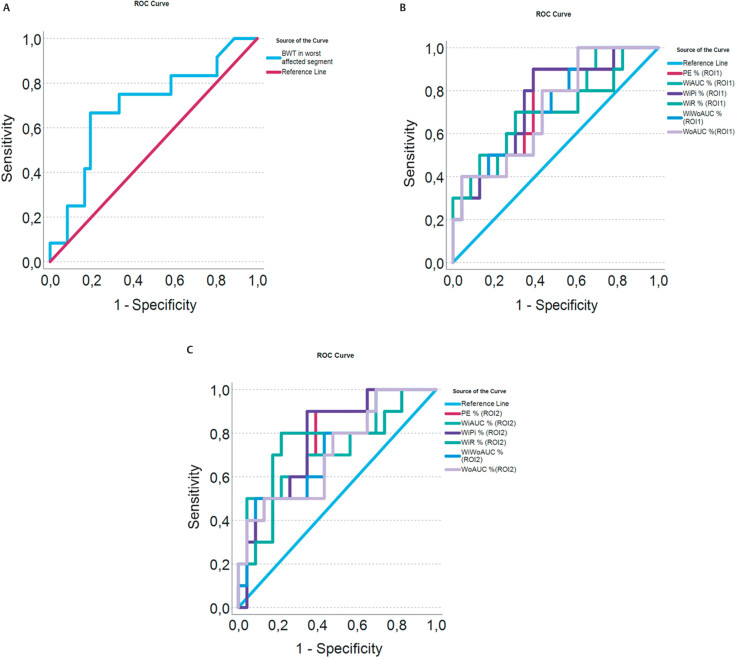
In panel **A** , a ROC-analysis is shown on comparing the bowel wall thickness in millimetres to the outcome moderate to severe inflammation on endoscopy. The AUROC was 0.70 (0.52–0.87). In panel **B** , a ROC analysis of ROI-1 is shown for all amplitude related DCE-US parameters such as PE, WiAUC, WoAUC, WiWoAUC, WiR, WoR and WiPI. For these parameters, the following AUROCs were found: PE%=0.74 (0.55–0.92), WiAUC%=0.73 (0.54–0.92), WoAUC%=0.72 (0.53–0.90), WiWoAUC%=0.73 (0.54–0.91), WiR%=0.69 (0.48–0.90) and WiPI%=0.75 (0.57–0.93). In panel **C** , a ROC analysis was performed for the same parameters for ROI-2. In this analysis, the AUROCs were as follows: PE%=0.77 (0.60–0.93), WiAUC%=0.73 (0.53–0.92), W0AUC%=0.71 (0.51–0.90), WiWoAUC%=0.72 (0.53–0.91), WiR%=0.74 (0.55–0.94) and WiPi%=0.77 (0.60–0.94). AUROC, area under the ROC curve; PE, peak enhancement; ROC, receiver operating characteristic; WiAUC, wash-in area under the curve; WiPI, wash-in perfusion index; WiR, wash-in rate; WoAUC, wash-out area under the curve; WoR, wash-out rate.

In a ROC analysis, the AUROC for BWT was 0.70 (0.52–0.87). The optimal cut-off with Youden’s index was 6.5 mm (sensitivity 67% and specificity 81%) for detecting moderate/severe disease. This analysis was performed for all significant DCE-US parameters (see Fig. 4 ). Of these, PE% for ROI1 had an AUROC of 0.74 (0.56–0.92). The optimal cut-off with Youden’s index was 27% (sensitivity of 90% and specificity of 61%) for detecting moderate/severe disease.

When comparing ROI-1 with ROI-2, the values from ROI-2 were significantly higher for all parameters ( *p* <0.001) except for the temporal parameters TTP ( *p* =0.003), RT ( *p* =0.001), mean transit time (MTT; *p* =0.049) and FT ( *p* =0.001) that were all significantly lower. The quality of fit between the data and the Vuebox model was significantly higher for ROI-1 versus ROI-2 (see the table in Appendix)

## Discussion

The feasibility of DCE-US analysis in patients with CD and a thickened bowel wall was 73%. A correlation was identified between the local SES-CD and the parameters PE and WiPI derived from the time–intensity curve (TIC) when they were normalised internally. Furthermore, there was a significant difference in contrast enhancement between mild and moderate/severe disease activity for the amplitude parameters PE, WiAUC, WoAUC, WiWoAUC, as well as for WiR and WiPI, both of which have a temporal component. There was a significant difference in all perfusion parameters between ROI1 and ROI2.

Measuring BWT is an effective method for separating CD patients in remission and those with even mild disease activity. 22​ However, it is crucial to identify patients with moderate to severe disease activity as they are more likely to develop complications of CD. 23​ ​24​ In this study, we compared two different ROIs when measuring contrast enhancement in the GI wall. One ROI included the entire wall (ROI-1) while the other focused only on the mucosa and submucosa (ROI-2). Previous studies have shown that perfusion is higher in the mucosa and submucosa of the bowel than in the proper muscle. 13​ Perfusion equals the blood volume divided by the MTT. In our analysis, the amplitude-related parameters in the TIC are related to blood volume, while the temporal parameters correspond to the MTT. As expected, all amplitude-related parameters in ROI-2 were significantly higher than those in ROI-1, and the temporal parameters were lower. Although CD is a transmural disease, most of the inflammatory changes are in the mucosa and submucosa. Changes in perfusion may be easier to detect in these areas because the higher signal intensity is less susceptible to noise. In our study, we observed a trend toward a higher AUROC for some parameters in ROI-2. However, since ROI-1 is smaller, it is also more susceptible to noise. Despite this, the ROI-2 data from showed only a slightly poorer fit to the DCE-US model used in the in the Vuebox analysis (92 vs 93%).

Ripolles et al. and Freitas et al. showed that CEUS can distinguish mild from moderate to severe disease. 7​ ​10​ Ripolles used SonoVue and a Toshiba scanner, and the parameter derived from performing the contrast examination is a relative parameter recorded at PE by the following equation: ((brightness postcontrast-brightness precontrast)/brightness precontrast)×100. The endoscopic reference standard was based solely on the presence or absence of ulcers, and patients with superficial or deep ulcers were categorised as having moderate or severe disease activity. Based on previous research by the group, an enhancement of>47% was considered active disease. They found that CEUS was particularly useful in cases with a thickened bowel wall without significant colour Doppler enhancement and recommended not using contrast if both bowel wall thickening and colour Doppler enhancement were present.

Freitas et al. used SonoVue and a Hitachi scanner in a retrospective study. The contrast analysis was performed on the scanner using proprietary software (EZU-CH8) and a technique called motion compensated microbubble trace imaging. In this study, the decibel value of the PE was used. The data were not normalised. The reference standard was SES-CD, and the cut-off between inactive/mild disease and moderate/severe disease was SES-CD≥7. In this cohort, 30 of 50 patients had moderate to severe activity in the terminal ileum. Using ROC analysis, the AUROC was 0.80 (0.60–0.94) with a sensitivity of 71.4% and a specificity of 78.9% for detecting moderate/severe diseases at the optimal cut-off.

Ripolles used an unvalidated reference; the Freitas study is retrospective, and in both cases, the DCE-US technique is specific to certain ultrasound scanners and thus difficult to reproduce.

We found that none of the parameters investigated in this study were strong discriminators between inactive/mild disease and moderate/severe disease. A BWT cut-off of 6.5 mm had low sensitivity and moderate specificity for detecting moderate/severe disease, whereas a PE of≥27% (and the other significant DCE-US parameters) had high sensitivity but poor specificity.

The strength of our study lies in a validated reference standard and in commercially available software that can be integrated with current ultrasound hardware. It is a prospective study in which the investigators were blinded to the results of the GIUS and the endoscopic score, respectively. A limitation of our study is the small number of patients in a single-centre setting. None of the investigators of endoscopy or ultrasound was blinded to patient symptoms of laboratory values. This could introduce a bias, but not one limited to a single modality. The relatively low feasibility and labor-intensive nature of the DCE-US analysis limit the usefulness of this method in clinical practice. However, the normalisation procedure, combined with the strict quality criteria for DCE-US data analysis, is a promising approach to improve the quality of studies using contrast agents to assess blood flow in the GI wall. If similar software for perfusion analysis is standardised on US scanners, it may be of clinical use.

In conclusion, it is feasible to perform DCE-US on CD patients with active disease using Sonazoid, but with a substantial number of analysis and post-processing failures. The analysis method is cumbersome, and the study should be repeated in a larger cohort. The information derived from DCE-US could detect patients with moderate to severe disease activity in CD but is not clinically useful now.

## Supplementary material

Supplementary Material Zusatzmaterial
