# Supplementary material for: Contrast-enhanced Perfusion Measurements in Patients with Active Crohn’s Disease Using Sonazoid
Source: Ultrasound Int Open. 2026 Apr 8;12:a28309879. doi: 10.1055/a-2830-9879 (PMC13062680; doi:10.1055/a-2830-9879)
Supplement: Supplementary file 1 — Supplementary Material [file 10-1055-a-2830-9879_28335803.pdf]

Appendix:

Material and methods:

GIUS

The bowel was scanned in the longitudinal axis while swiping back and forth to include all parts of the GI tract in the investigation. When possible, to avoid measuring in empty bowel loops and overestimating bowel wall thickness, the transducer was moved to a bowel loop with faecal content, or the examiner waited for peristalsis to fill the bowel loop. The image plane was aligned parallel to the longitudinal axis of the bowel and perpendicular to the surface of the anterior wall to avoid oblique measurements and folds. The measurements were made in areas with a clear interface between the mucosa and the intestinal lumen. The wall layers corresponding to the mucosa, submucosa, and the proper muscle were measured using the zoom function to improve the accuracy of cursor placement. Two measurements were taken for each, and the mean value was used. Wall layers could not be measured in areas where stratification was lost. Due to an insufficient axial resolution of the C1-6 transducer, the wall layers of the rectum were not measured. If the BWT was ≥3 mm, measurements were taken in the thickest part of the segment. If multiple bowel segments were affected, the segment with the thickest bowel wall was considered the most severely affected and used for comparison with SES-CD.

Definitions:

Ultrasound remission was defined as BWT <3 mm. Ultrasound activity as BWT ≥ 3 mm.

Bowel wall thickness (BWT) and bowel wall layers: In the normal bowel wall, three layers are consistently visible with sufficient resolution of the ultrasound transducer. In the anterior bowel wall, the first hypoechoic layer corresponds to the proper muscle, the second hyperechoic layer to the submucosa, and the third hypoechoic layer to the mucosa, approximately. Sometimes, the interface echoes between the serosa and the proper muscle, and the mucosa and the lumen, are well defined. In these cases, you see five wall layers. These interface echoes are not always clear, however. Therefore, bowel wall thickness was measured in the anterior wall from the border of the interface between the serosa and the proper muscle to the border of the interface between the mucosa and the lumen. The individual bowel wall layers were measured by placing the cursor at the exact border between the layers.

Colour Doppler: The vessel density was graded from 0 to 2 as follows: 0-1 vessel/cm^2=0, 2-5 vessels/cm^2=1 and >5 vessels/cm^2=2. We also investigated the presence or absence of colour Doppler signals as a separate variable.

Stratification: If the layers corresponding to the mucosa, submucosa, and proper muscle were visible, the stratification was normal. Partial loss of stratification was defined as limited disruption of the layers within the affected segment, whereas total loss of stratification was defined as diffuse loss throughout the entire segment. We investigated both the grading of stratification loss and the presence of stratification loss.

Fatty wrapping: Presence of fat wrapping was registered and defined as a hyperechoic mass encasing at least half the circumference of the bowel.

Folds in the proper muscle: The proper muscle should form a straight line in the longitudinal direction, as it can only shorten during contraction or lengthen during relaxation. If the proper muscle was folded, this was registered.

Thickening of the muscularis mucosa: If two extra ultrasound layers could be seen between the hyperechoic submucosal layer and the hypoechoic mucosal layer, the segment was categorised as having a thickened muscularis mucosa. This is a feature of Crohn's disease that is related to the development of fibrosis (35).

Compressibility: A bowel segment typically deforms/changes configuration when compressed with the ultrasound transducer. If there was no visible deformation of the bowel wall segment during compression with the ultrasound transducer, it was considered non-compressible.

Complications: A presence of stenosis was registered and defined as a thickened wall with a narrowing of the lumen with or without prestenotic dilatation, with lumen >2.5 cm. Stenoses were also graded as present, with or without prestenotic dilatation. Presence of fistulas was registered and defined as a hypoechoic tract with or without bright spots between two lumens or to the surface. We investigated the presence of complications, the grading of stenosis, the presence of stenosis, and the presence of fistulas as separate variables.

DCE-US: examination and analysis

The C1-6 ultrasound transducer was used for the examination with a preset specifically designed for Sonazoid, with a mechanical index of 0.26-0.28. The mechanical index was set by adjusting power and depth. The examination was recorded in dual mode, with an image displaying the B-mode information in the left panel and contrast information in the right panel. Focus was set at the lower border of the anterior bowel wall. The gain was reduced so that only minor background signals from the tissue were visible in contrast mode before the injection.

The transducer was oriented in the longitudinal direction and perpendicular to the bowel wall segment. The ultrasound transducer was kept as stationary as possible during the acquisition of the contrast examination. Some bowel segments were longer than the section covered by the transducer, and therefore, the examiner, blinded to the endoscopy, could not be certain that the two areas corresponded exactly.

The injection of 1 mL Sonazoid was performed over 2 seconds followed immediately by the injection of 10 mL of 0.9% saline over 4 seconds. The area was then recorded for 90 seconds before the cine loop was saved.

The DICOM files were individually uploaded to Vuebox and down-sampled to 5 Hz to reduce the file size and facilitate analysis in the software. Then, an area of interest was drawn around the anterior wall in the affected bowel, and this region was stabilized using onboard motion correction, with the image with peak enhancement as the reference (Figure 1). Out-of-plane images were removed. Two regions of interest (ROI) were drawn in the bowel wall closest to the probe within the area of interest. ROI-1 included the full wall thickness, and ROI-2 only the mucosa and submucosa. The ROIs were drawn freehand following the shape of the bowel wall or the bowel wall layers. The size of the ROIs varied according to the size of the inflamed bowel segment that could be included in sector of the transducer. If a part of the bowel in the segment was normal, it was not included in the ROIs. Then the software analysed the contrast intensity of these regions.

Even though Vuebox re-linearises the data, it still needs to be normalised. The primary reason for normalising the data is that the attenuation varies from patient to patient (25). The normalisation is needed to adjust for this. In this study, we normalised the data to the contrast intensity inside a bowel wall vessel. After analysing the cine loop, the software constructs a heat map of the enhancement within the stabilized regions of interest. In this heatmap, vessels within the bowel wall stand out as “hot spots”. Within these hot spots, the blood volume is 100%. This can be used to normalise the contrast data as the intensity values in the vessel “hot spot” is used as a reference for the corresponding values in ROI-1 and ROI-2 (Figure 2).

The following parameters can be derived from Vuebox: Temporal parameters such as time to peak (TTP), rise time (RT), fall time (FT), and mean transit time (MTT). Amplitude parameters such as peak enhancement (PE), wash-in area under the curve (WiAUC), wash-out area under the curve (WoAUC), and wash-in and wash-out area under the curve (WiWoAUC). Finally, combined parameters such as wash-in rate (WiR), wash-out rate (WoR), and Wash-in perfusion index (WiPI). All parameters were collected as linear data, and the amplitude and combined parameters were normalised.

Both the linear and normalised parameters derived from the time-intensity curve were correlated with the SES-CD value for the bowel segment included in the contrast examination. The quality of fit between the contrast data and the model used in Vuebox was calculated, and a value over over 80% was considered acceptable. Feasibility was defined as the percentage of patients without technical failures and acceptable quality of fit.

Thieme

Table: Linear DCE-US parameters

Parameters	ROI-1			ROI-2		
	Remission or mild disease (SES-CD<7)[1] (n=23)	Moderate or severe disease (n=10)	P[2]	Remission or mild disease (SES-CD<7)[1] (n=23)	Moderate or severe disease (n=10)	P[2]
Time to peak (s)	8.6 (1.6)	8.2 (1.8)	0.743	8.4 (1.9)	8.0 (1.9)	0.658
Rise time (s)	5.5 (1.6)	5.0 (1.2)	0.451	5.4 (2.1)	4.8 (1.3)	0.343
Fall time (s)	12.0 (4.6)	10.6 (4.2)	0.237	11.5 (4.9)	9.8 (4.3)	0.167
Mean transit time (s)	21.3 (20.5)	21.1 (21.4)	0.923	21.2 (15.6)	21.8 (18.7)	0.923
Peak enhancement (au)	2037 (4006)	1155 (1126)	0.576	2552 (4175)	1578 (1582)	0.576
Wash-in area under the curve (au)	5724 (11432)	3390 (3647)	0.363	8041 (14778)	4964 (5258)	0.451
Wash-out area under the curve (au)	11755 (22424)	6632 (7014)	0.305	16692 (26308)	9737 (10140)	0.343
Was in and wash-out area under the curve (au)	17480 (36953)	9987 (10661)	0.305	25245 (39472)	14768 (15398)	0.384
Wash-in rate (au/s)	444 (1096)	328 (344)	0.714	553 (1533)	474 (481)	0.802
Wash-out rate (au/s)	154 (400)	122 (131)	0.862	194 (454)	167 (179)	0.954
Wash-in perfusion index (au/s)	1305 (2525)	745 (720)	0.576	1641 (2718)	1018 (1020)	0.576

[1] Median (interquartile range), [2] p-value for Mann Whitney U-test

Table: Comparison of measurements in Region of interest 1 versus Region of interest 2.

Parameter	ROI-1	ROI-2	p-value
Peak enhancement (au)[1]	28.2 (12.2)	35.3 (17.3)	**<0.001**
Wash-in area under the curve (au)[1]	32.6 (17.2)	39.7 (25.2)	**<0.001**
Wash-out area under the curve (au)[2]	40.9 (20.1)	50.7 (25.0)	**<0.001**
Wash-in wash-out area under the curve(au)[2]	39.0 (17.6)	48.4 (22.1)	**<0.001**
Wash-in rate(au/s)[2]	25.8 (12.5)	33.3 (20.1)	**<0.001**
Wash-out rate(au/s)[2]	25.0 (15.1)	33.0 (21.3)	**<0.001**
Wash in perfusion index (au/s)[1]	29.1 (12.6)	34.8 (16.4)	**<0.001**
Time to peak (s)[1]	8.12 (1.9)	8.06 (2.0)	**0.003**
Rise time(s)[1]	5.4 (1.7)	5.3 (1.9)	**0.001**
Fall time(s)[1]	11.6 (4.7)	10.9 (4.5)	**0.001**
Mean transit time(s)[1]	21.3 (21.3)	21.2 (16.4)	**0.049**
Quality of fit (%)[2]	93.0 (4.4)	92.0 (5.2)	**0.044**

[1] Wilcoxon Signed Rank test [2] Paired sample T-test
